# Cystic Angiomyofibroblastoma of the Uterus Mimicking Ovarian Cancer

**DOI:** 10.3390/medicina60101645

**Published:** 2024-10-08

**Authors:** Jae Yoon Jo, Hyo Jung An, In Ae Jo, Jeong Kyu Shin, Won Jun Choi, Jong Chul Baek

**Affiliations:** 1 Department of Obstetrics and Gynecology, Gyeongsang National University School of Medicine, Gyeongsang National University Hospital, Jinju 52727, Republic of Korea; poodeeng@naver.com (J.Y.J.); obgychoia@gnu.ac.kr (I.A.J.); 2848049@hanmail.net (J.K.S.); choiwj@gnu.ac.kr (W.J.C.); 2 Institute of Medical Science, Gyeongsang National University, Jinju 52727, Republic of Korea; ariel2020@naver.com; 3 Department of Pathology, Gyeongsang National University School of Medicine, Gyeongsang National University Changwon Hospital, 11, Changwon-si 51472, Republic of Korea; 4 Department of Obstetrics and Gynecology, Gyeongsang National University School of Medicine, Gyeongsang National University Changwon Hospital, 11, Changwon-si 51472, Republic of Korea

**Keywords:** angiomyofibroblastoma, uterus, breast cancer, prognosis

## Abstract

Angiomyofibroblastoma (AMFB) is an exceedingly rare mesenchymal tumor of the lower genital tract. AMFB primarily affects the pelviperineal region, especially the vulvar in premenopausal women. Typically, AMFB is a benign disease and does not have the potential for metastasis or recurrence, requiring complete surgical excision. Its accurate differentiation from aggressive angiomyxoma is critical due to varying prognoses. A 51-year-old woman, diagnosed with mucinous carcinoma of the breast, presented with a 12 cm abdominopelvic mass identified during breast cancer staging. Imaging suggested an ovarian origin; however, surgical exploration revealed a stalk-attached cystic mass in the anterior body of the uterus. Histopathology confirmed AMFB. Immunohistochemical analysis showed positivity for estrogen and progesterone receptors and smooth muscle actin. The patient continued breast cancer treatment postoperatively without pelvic mass recurrence or complications for a postoperative follow-up period of one year. This case highlights AMFB’s potential uterine body origin, expending known tumor sites and complicating diagnosis due to overlapping features with other mesenchymal tumors. Accurate diagnosis using immunohistochemical markers and pathological features is essential to avoid unnecessary aggressive treatments. The uterine location in this case suggests a possible shared pathogenesis with uterine myomas, warranting further research into their connection. Reporting the first case of AMFB originating in the uterine body enhances understanding of this rare condition and underscores the importance of clinical awareness and precise diagnostic strategies to guide management and improve outcomes.

## 1. Introduction

AMFB is an exceedingly rare condition, typically encountered only through case reports. It was first described as a mesenchymal tumor by Fletcher et al. in 1992 [1]. Predominantly affecting the external genital organs such as the vulva and vaginal opening in premenopausal women, there have been approximately 200 case reports to date, including cases reported in men [2,3]. As previously mentioned, this tumor predominantly arises in the vulva, although reports have emerged of it originating within the pelvis. They were located in the vagina in about 10–15% of cases. To our knowledge, four cases have been published in English concerning AMFB arising from the broad ligament and fallopian tube in premenopausal women [4,5,6]. Most AMFBs were localized, benign tumors that do not recur or metastasize, making complete surgical excision the treatment of choice. However, due to a case report of sarcomatous transformation, diligent follow-up is necessary [7]. Furthermore, it is imperative to distinguish AMFB from aggressive angiomyxoma, as they share similar clinicopathological characteristics but differ in prognosis. Since their immunohistochemical features are alike, histological differentiation remains the most definitively established method [1,8].

We present what we believe to be the first reported case of AMFB originating in the uterine body and reviewed series of uterine cervical AMFB cases. Helpful imaging characteristics in these entities and potential pathogenesis were discussed.

## 2. Case Report

**Figure 1:**
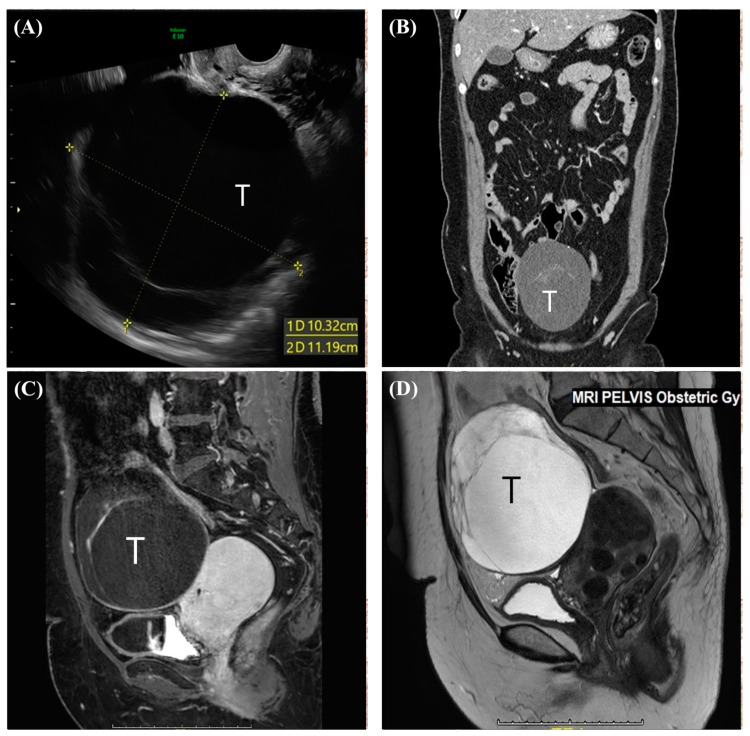
(**A**) Transvaginal ultrasonographic image of pelvic cavity showing a right adnexal cystic mass with septation. (**B**) Contrast-enhanced CT in the delayed phase (coronary view) showing a 12 cm abdominopelvic cystic mass with a thick septum which located abdominopelvic cavity. (**C**) T1-weighted magnetic resonance image (Sagittal view) with tumor (T) showing slightly hypointense compared to skeletal muscle and enhanced by gadolinium at septum. (**D**) T2-weighted magnetic resonance image (Sagittal view) with tumor (T) showing a hyperintense mass with a curvilinear dark signal intensity.

A 51-year-old unmarried woman, gravida 0, consulted a gynecologic oncologist. She was diagnosed with mucinous carcinoma breast cancer via a core biopsy conducted four months prior to her obstetrics and gynecology consultation. She was on medication for hypertension in addition to breast cancer and had a history of undergoing a myomectomy for uterine myoma a decade ago. Her father was diagnosed with prostate cancer, and her mother underwent treatment for lung cancer. Transvaginal ultrasound revealed a right cystic adnexal mass with multiple septations, measuring approximately 11.2 cm and 10.3 cm at their widest points (Figure 1A). During the staging workup for breast cancer, a CT scan revealed a 12 cm abdominopelvic cystic mass with a thick septum, suspected to originate from the right ovary, located behind the bladder and in front of the uterus (Figure 1B), leading to referral to gynecology. On pelvic MRI, the T1-weighted image revealed the mass as slightly hypointense compared to skeletal muscle and enhanced by gadolinium (Figure 1C). The T2-weighted image showed a hyperintense mass with a curvilinear dark signal intensity, raising suspicion of borderline ovarian cancer (Figure 1D). She experienced no pelvic pain or vaginal bleeding related to the pelvic mass and had never been gynecological exam before. Pelvic examination revealed a firm, mobile, round mass without tenderness. The patient was menopause last year, with pre-surgery serum FSH/E2 levels at 170 mIU/15 pg/mL, and the ROMA (ovarian malignancy risk algorithm) test results for an epithelial ovarian cancer risk showed CA125 at 6.89 U/mL and HE4 at 50.40 pmol, indicating a low risk of postmenopausal epithelial ovarian cancer. She underwent four sessions of neoadjuvant chemotherapy (doxorubicin and cyclophosphamide) and underwent breast-conserving surgery four months after her breast cancer diagnosis. The sentinel lymph node frozen biopsy reported negative results; thus, the axillary lymph node dissection was not performed. Total laparoscopic hysterectomy with both salpingo-oophorectomy (TLH c BSO) was also planned to be performed concurrently with the breast operation.

**Figure 2:**
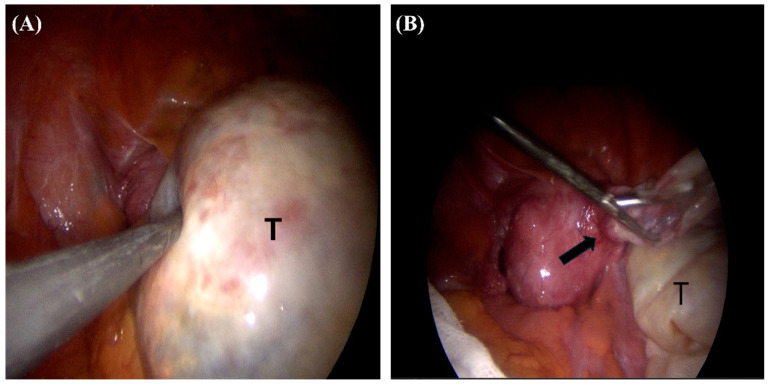
Intraoperative appearances: (**A**) the cystic tumor (T) located in the right pelvis; (**B**) the tumor after cyst aspiration showing an origin at the body of the uterus with a stalk (black arrow).

Contrary to expectations of ovarian origin, the multiseptated cystic tumor attached to the anterior uterine body with a stalk. The cystic mass was resected after aspiration and sent for pathologic examination (Figure 2A,B). Frozen biopsy showed benign disease entities. The uterus displayed multiple intramural myomas, and both ovaries appeared grossly intact. TLH c BSO was performed. She was discharged without complications on the 9th postoperative day. The biopsy of the left breast cancer revealed mucinous carcinoma, mixed type, histologic grade I, with a mitotic count of one. There was no lymph node metastasis, and ER and PR were positive, but HER2 was negative.

**Figure 3:**
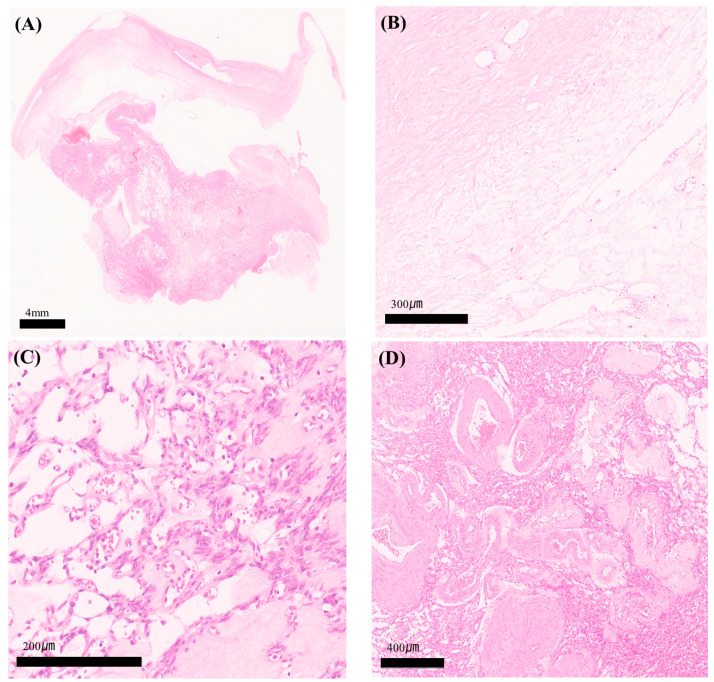
Hematoxylin and eosin (H&E) staining of specimen. (**A**) A relatively well-circumscribed multicystic tumor consisting of hypocellular and hypercellular lesions (H&E staining; magnification: ×10). (**B**) In the hypocellular lesion, the stroma was hyalinized and had an edematous appearance (H&E staining; magnification: ×200). (**C**) In the hypercellular lesion, spindle or oval-shaped stromal cells were oriented and aggregated around thin-walled vessels (H&E staining; magnification: ×200). These tumor cells had bland-looking chromatin and inconspicuous nucleoli without mitosis. (**D**) Occasionally, larger engorged vessels were intermingled with the tumor cells (H&E staining; magnification: ×100).

The pathology of the uterus and ovaries showed no abnormalities other than multiple intramural myomas. The outer surface of the cystic mass was whitish and glistening grossly. The tumor was 13 × 7 × 2.5 cm in total. The cut surface appeared whitish flesh-like color with multi-septation and multi-cystic as well as intra-tumoral hemorrhage. In the microscopy image, thge representative section showed a relatively well-circumscribed multicystic tumor, with alternating hypocellular and hypocellular lesions (Figure 3A). In the hypocellular lesion, the stroma was hyalinized and had an edematous appearance (Figure 3B). On the contrary, in the hypercellular lesion, spindle or oval-shaped stromal cells were oriented and aggregated around thin-walled vessels (Figure 3C). These tumor cells had bland-looking chromatin and inconspicuous nucleoli. Mitoses were absent. Occasionally, thick-walled engorged vessels were intermingled with the tumor cells (Figure 3D).

**Figure 4:**
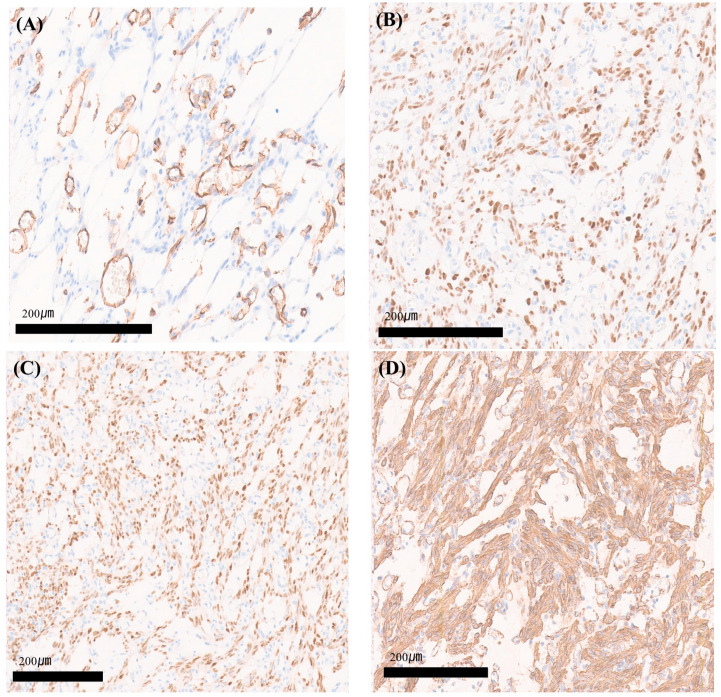
(**A**) Intra-tumoral capillary-sized vessels were positive for CD31, a vascular maker. However, tumor cells were negative for CD31 (original magnification: ×200). (**B**) Tumor cells were positive for the estrogen receptor (ER) (original magnification: ×200). (**C**) Tumor cells were positive for the progesterone receptor (PR) (original magnification: ×200). (**D**) Tumor cells were positive for smooth muscle actin (SMA) (original magnification: ×200).

Immunohistochemically, intra-tumoral capillary-sized vessels were positive for CD31, a vascular maker. However, tumor cells were negative for CD31. This suggested that the tumor cells were neither endothelial cells nor of vascular origin (Figure 4A). Tumor cells were positive for estrogen (Figure 4B) and progesterone receptors (Figure 4C), showing strong expression with smooth muscle actin (Figure 4D). Tumor cells were negative for CD34 and D2–40 and focally positive for Desmin. The exophytic mass attached to the anterior uterine body was diagnosed as angiomyofibroblastoma. 

She underwent adjuvant chemotherapy and radiotherapy for breast cancer without complications or recurrence for 12 months postoperatively.

## 3. Discussion

Angiomyofibroblastoma (AMFB) occurs in middle-aged premenopausal women and typically presents in the vulva, often leading to a clinical diagnosis of Bartholin’s cyst. AMFB is a rare, benign mesenchymal tumor requiring further study to understand its pathophysiology. Fletcher et al., who first described this tumor, noted it as a mixed echoic soft tissue tumor, sometimes containing cystic spaces, which potentially correlates with cellularity [1]. AMFB is usually less than 5 cm in size. It appears most common in women of reproductive age. Patients typically present with a painless mass that is misinterpreted as a cyst. Clinically, it usually presents as a slow-growing, painless subcutaneous mass. Although it primarily occurs in the external genital area, its clinical symptoms are nonspecific and uncommon, making location-based diagnosis challenging. If it is pedunculated in some cases, it might cause pain [9]. Although this rare mesenchymal tumor occurs primarily in the external genital area, there have been seven reports in the English literature of AMFB that have presented in uterine cervix so far (Table 1). 

Figueiredo et al. stated that MRI often shows myxoid tissue in angioid mesenchymal tumors, appearing as hyperintense with enhancement on T2-weighted images compared to muscle [17]. Ultrasound and physical examinations are able to exclude and classify advanced pelvic tumors to guide treatment decisions. However, when anatomical structures are difficult to discern or the tumor is exceptionally rare, additional MRI scans can provide a more precise understanding of the anatomy, thereby narrowing down the suspected diagnosis. MRI findings of AMFB note that while there may be potential to differentiate it from other tumors, achieving an accurate diagnosis remains challenging and there is currently no consensus on the typical findings of AMFB on MRI or USG [18].

AMFB, alongside aggressive angiomyxoma and cellular angiofibroma, exhibits overlapping features differing clinical behaviors as mesenchymal neoplasms. Aggressive angiomyxoma (AAM) and angiomyofibroblastoma (AMFB) are rare mesenchymal tumors with overlapping clinicopathological characteristics [19]. In certain cases, the differential diagnosis between the two tumors can be difficult, even for the most experienced pathologists [20]. Accurate diagnosis is essential to anticipate the behavior of these neoplasms and ensure appropriate clinical management. The gross appearance of AMFB is characterized by a well-demarcated edge and a thin pseudo-capsule surrounding the tumor. The cut surface of the tumor was tan-white and firm [9]. Immunohistochemical markers such as ER, PR, CD34, smooth muscle actin, and Desmin are used for differentiation, but overlapping characteristics render this unclear. Nonetheless, it is crucial to distinguish it from aggressive angiomyxoma, which requires wide excision. Even with surgical management, there is a 40–70% recurrence rate, necessitating regular follow-ups. Misclassifying AMFB as aggressive angiomyxoma can lead to unnecessary wide excisions, increasing patient morbidity and complications.

This patient had a myomectomy 10 years ago and presented multiple intramural myomas in the uterus following a hysterectomy. The uterine location of the AMFB and slightly elevated E2 levels despite menopause would suggest a possible shared pathogenesis with uterine myomas. Moreover, several previous case reports documented AMFB in female genital organs in patients taking tamoxifen [11,15]. This may also be considered as evidence supporting the possibility that hormonal stimuli participate in the pathogenesis of AMFB in female genital organs. The histogenesis of AMFB remains uncertain, and the diagnostic molecular pathology is not of clinical relevance. However, some suggested it originates from primitive mesenchymal cells, progressing through myoid differentiation to myofibroblasts under various microenvironmental and/or hormonal stimuli [1,18,21]. Aberrant expression of the HMGI-C gene has been found in a wide range of benign mesenchymal tumors. Horiguchi et al. suggested that abnormal expression of high mobility group I-C (HMGI-C) is involved in the pathogenesis of AMFB [22]. Kurose et al. found aberrant alternative splicing of HMGI in uterine fibroids, indicating a need for further research on the connection between uterine myomas and AMFB [21]. The deregulated expression of HMGI-C has been implicated in the tumorigeneses of AMFB, although the underlying mechanism of HMGI-C expression needs to be further investigated [23,24].

## 4. Conclusions

AMFB is an exceedingly rare condition, typically occurring in external genitalia, making cases arising in the pelvic cavity even more challenging to suspect. We reported the first instance of AMFB originating from the uterine body, which would enhance the literature in such rare cases and aid in developing more effective diagnostic and therapeutic approaches. Moreover, the distinctive presentation of AMFB in the uterus has broadened the understanding of potential tumor sites and underscored the need for clinical awareness.

## Figures and Tables

**Table 1:** Cases of AMFB diagnosed in the cervix of the uterus presenting with the clinical information.

Case	Age	Size (cm)	Presentation	Para	Treatment	Outcome	IHC	Reference
1	44	2	Polypoid mass	ND	ND	ND	desmin/vimentin (+/+), E/P (ND/ND) αSMA/CD34 (ND/ND), S100 (ND)	Babala, P., et al. [10]
2 *	43	3 × 3 × 2.5	No Sx.	3	Local excision	ND	desmin/vimentin (+/+), E/P (+/+) αSMA/CD34 (−/−), S100 (ND)	Kim, M.J., et al. [11]
3	53	4 × 3	Vaginal mass	3	LAVH & BSO	2 years	desmin/vimentin (+/ND), E/P (ND/ND) αSMA/CD34 (+/+), S100 (ND)	Lee, C., et al. [12]
4	32	1.2	Vaginal spotting	ND	Local excision	ND	desmin/vimentin (−/ND), E/P (+/+)αSMA/CD34 (ND/−), S100 (-)	Wong, Y.P., et al. [13]
5	48	1	Vaginal spotting	ND	Local excision(conization)	ND	desmin/vimentin (+/ND), E/P (+/+) αSMA/CD34 (+/+), S100 (ND)	Roncati, L., et al. [14]
6 *	40	6 × 5	Vaginal bleeding	ND	TAH & BSO	ND	desmin/vimentin (+/+), E/P (+/+) αSMA/CD34 (−/−), S100 (ND)	Büyüktalancı, D.Ö., et al. [15]
7	45	2.5 × 2 × 1	AUB	ND	Local excision	ND	desmin/vimentin (+/+), E/P (+/+) αSMA/CD34 (ND/+), S100 (ND)	Güvendi, G.F., et al. [16]

* Case with breast cancer history; IHC—immunohistochemistry; +—positive staining; −—negative staining; ND—not described; LAVH & BSO—laparoscopic-assisted vaginal hysterectomy and bilateral salpingo-oophorectomy; TAH—total abdominal hysterectomy; AUB—abnormal uterine bleeding; E—estrogen receptor; P—progesterone receptor.

## Data Availability

The datasets used and/or analyzed during the current study are available from the corresponding author upon reasonable request.
